# Benzodiazepine Consumption, Functionality, Cognition, and Somnolence in Older Adults at a Tertiary Care Psychiatric Hospital in Mexico City

**DOI:** 10.7759/cureus.53252

**Published:** 2024-01-30

**Authors:** Adolfo Montes-Castrejon, Luis Gerardo Moncayo-Samperio, Monica Flores-Ramos

**Affiliations:** 1 Department of Education, National Institute of Psychiatry, Mexico City, MEX; 2 Division of Postgraduate Studies, National Autonomous University of Mexico, Mexico City, MEX; 3 Department of Psychogeriatrics, National Institute of Psychiatry, Mexico City, MEX; 4 Department of Clinical Research, National Institute of Psychiatry, Mexico City, MEX

**Keywords:** excessive daytime sleepiness, functional capacity of the elderly, montreal cognitive assessment, cognition disorders, benzodiazepine use, cognitive impairment and dementia, cognition impairment

## Abstract

Background: The aging population in Mexico, particularly those aged 60 and above, faces challenges in healthcare, including potentially inappropriate prescriptions of benzodiazepines. Physiological changes in older adults make precise drug prescriptions crucial.

Objective: This study aims to evaluate and compare functionality, cognition, and daytime somnolence in older adults using benzodiazepines versus non-users. Additionally, it outlines the demographic and clinical characteristics of both groups.

Methods: A cross-sectional study enrolled 162 participants aged 60 and above, categorized as benzodiazepine consumers or non-consumers. Assessment tools included Lawton’s Index, Montreal Cognitive Assessment, Epworth Sleepiness Scale, and Benzodiazepine Dependence Questionnaire. Statistical analysis employed t-tests and chi-square tests.

Results: Benzodiazepine users (n=81) exhibited lower cognitive scores, increased sleepiness, and reduced daily living activities compared to non-users (n=81). Demographically, BZD users had lower education levels.

Conclusion: Benzodiazepine use in older adults is associated with cognitive decline, daytime somnolence, and functional limitations, emphasizing the need for cautious prescription practices and continual monitoring. This study contributes insights into the impact of benzodiazepines on the cognitive health of older adults in Mexico.

## Introduction

Background

The demographic landscape in Mexico is undergoing significant changes, especially in the population over 60 years of age. It's projected that this demographic will increase from 6.3% of the total population in 2010 to nearly 23% by 2050 [1]. With the total population expected to reach 150 million by 2050, the 65 years and older group is nearing 28.7 million [1].

When prescribing medication to older adults, it's critical to consider the need for clear indications for pharmacological treatment and the risks of potentially inappropriate prescriptions. These risks stem from physiological changes in older adults, such as reduced first-pass hepatic metabolism leading to higher concentrations of liver-metabolized drugs, increased body fat mass affecting the distribution and half-life of lipophilic drugs, and decreased total body water influencing the concentration of water-soluble drugs [2]. Therefore, careful evaluation of drug prescriptions in older adults is vital to avoid potentially inappropriate prescriptions.

Benzodiazepines are a case in point, often deemed potentially inappropriate due to their link with increased morbidity, hospitalization, and higher healthcare costs [3]. Along with non-steroidal anti-inflammatory drugs, benzodiazepines are among the most inappropriately prescribed medications worldwide [4].

Pharmacology of benzodiazepines

Benzodiazepines, absorbed fully upon administration, are categorized by their elimination half-life into ultra-short, short, intermediate, and long-acting groups. They strongly bind to plasma proteins, with variations in binding levels among different drugs. Notably, in older adults, reduced serum albumin may elevate the active, unbound drug fraction, intensifying effects [5]. Metabolized primarily in the liver by enzymes like CYP3A4, certain benzodiazepines, like lorazepam, bypass these enzymes. Drug metabolism can be affected by CYP3A4 inhibitors [5]. Large distribution volumes, increasing with age, may result in prolonged drug retention and heightened sedation [6]. Pharmacodynamically, benzodiazepines enhance the inhibitory effect of gamma-aminobutyric acid (GABA) at the GABA-A receptor, leading to increased chloride channel conductance, crucial for their anxiolytic and sedative-hypnotic effects [7].

Epidemiology and use of benzodiazepines in older adults

Benzodiazepines are widely prescribed in the United States, with around 92 million prescriptions dispensed in pharmacies in 2019, with alprazolam accounting for 38% of these sales [7]. The 2017 National Drug Use Survey in Mexico revealed that 0.9% of individuals aged 12 to 65 years have used tranquilizers, including benzodiazepines. It’s important to note that this statistic is particularly focused on the 35-to-65-year-old segment of the population. This usage is the sole instance reported in the study’s aggregated epidemiological data [8].

Indications for benzodiazepines in older adults

Approved by the U.S. Food and Drug Administration, benzodiazepines are prescribed to treat conditions such as generalized anxiety disorder, insomnia, epilepsy, social phobia, and panic disorder [7]. However, after a wide search, no other specific medical indications exclusively tailored to older adults for the use of benzodiazepines were found in this population.

The 2019 Canadian Guidelines on Benzodiazepine Disorder in Older Adults discourage the first-line use of benzodiazepines for treating insomnia and anxiety disorders. Instead, cognitive-behavioral therapies (CBT) are recommended, supported by moderate to strong evidence of their effectiveness. Benzodiazepines should only be considered after exploring other pharmacological options, such as antidepressants, and always at appropriate doses and durations. Strong evidence underlines the importance of informing older adults about the benefits, risks, and alternatives before considering benzodiazepines. The guidelines advocate for short-term benzodiazepine use, not exceeding four weeks, and minimum effective doses. Additionally, it is recommended to assess prior substance use and potential use disorders to prevent the development of benzodiazepine use disorder, with strong evidence supporting this precautionary measure [9].

Chronic use, benzodiazepine use disorder and functionality in older adults

Studies have identified determinants for chronic benzodiazepine use (≥ 180 days) in older adults, including being female, having public health insurance, experiencing depressive episodes, sleeping less than six hours per night, suffering from hypertension, joint pain, poor perceived health status, and tobacco use. Living alone was identified as a protective factor against chronic use [10].

Benzodiazepine use disorder, as defined in the Diagnostic and Statistical Manual of Mental Disorders, Fifth Edition (DSM-5) encompasses a range of problematic behaviors associated with the use of these drugs. The DSM-5 criteria include patterns of excessive use, inability to control usage, time spent in obtaining the drug, cravings, and use despite adverse consequences [11]. Diagnosis depends on the number of criteria present, ranging from mild to severe.

In older adults, the Canadian guidelines emphasize the importance of a thorough evaluation, considering factors such as age of onset, drug type, dosage, and use pattern, as well as the patient's overall health and social environment [9]. Dependence is typically associated with longer-term use and higher dosages and can manifest in various symptoms, including withdrawal and cognitive effects like confusion [12].

Moreover, the chronic use of benzodiazepines in older adults has been linked to reduced functionality, as these drugs can significantly affect daily living activities and mobility. Studies in France have demonstrated that benzodiazepine users face increased limitations in daily activities and mobility, irrespective of the duration of action of the drug or the indication for its use [13]. The World Health Organization highlights the importance of considering a person's interaction with their environment when assessing functionality, suggesting a cautious approach to benzodiazepine use in older adults to prevent physical disability [14].

Benzodiazepines, cognition, and sleepiness in older adults

Research has linked benzodiazepine (BZD) use with dementia in older adults, though it remains a contentious issue. A systematic review indicated an association between BZD use and increased dementia risk, especially with long-acting agents, daily use over three years, and previous use, though a causal relationship wasn't definitively established [15]. A Spanish study reported a higher risk of dementia among BZD users, particularly with short to intermediate half-life BZDs, but the risk was not significant after adjusting for various health and demographic factors [16]. Another study among U.S. veterans found that cumulative exposure to BZDs was only minimally associated with increased dementia risk [17].

Excessive daytime sleepiness (EDS), a known side effect of BZDs, has been studied for its impact on older adults. A meta-analysis showed that BZD treatment increased the likelihood of EDS compared to placebo [18].

In Latin America, a high prevalence of BZD use among older adults has been reported, with diazepam being the most commonly used for treating anxiety and insomnia. However, no significant correlation between BZD use and cognitive impairment in older adults was found in the region [19]. Research in Mexico by Minaya et al. at the National Institute of Psychiatry Ramon de la Fuente Muñiz highlighted that BZD dependence was associated with more severe depression and anxiety symptoms and lower cognitive and psychosocial functioning [20].

This study investigates the impact of benzodiazepine use on older adults, hypothesizing that individuals taking these medications will show reduced functionality and cognition, along with increased daytime somnolence, compared to non-consumers. The primary objective is to evaluate and compare functionality, cognition, and daytime somnolence in older adults, distinguishing between those using benzodiazepines and those who are not. Additionally, the study aims to outline and compare demographic and clinical characteristics between the two groups of older adults.

## Materials and methods

This study enrolled individuals aged 60 and above, categorizing them as either benzodiazepine consumers or non-consumers based on specific criteria. Inclusion criteria for benzodiazepine consumers involved being literate outpatients at the National Institute of Psychiatry Ramon de la Fuente Muñiz (INPRFM), a tertiary care institution, actively using benzodiazepines. Non-consumers, also aged over 60 and literate, were either INPRFM patients or relatives of patients, without active benzodiazepine use. Exclusion criteria for both groups included chronic-degenerative diseases, severe psychiatric diagnoses, or intellectual disability for benzodiazepine consumers, and the additional criterion of current benzodiazepine use for non-consumers. Participants unable to complete the required study instruments were excluded.

The study population comprised elderly patients from the outpatient clinic of INPRFM in Mexico City. A sample size of 162 subjects (81 per group) was determined using a two-mean comparison formula based on an effect size of 0.3, an alpha error of 0.05, a beta error of 0.85, a standard deviation of 5, and a critical χ² of 11.07.

The measurement instruments used in the study included assessments such as the Lawton's Index, which evaluates eight daily tasks and scores from 0 to 8. A score of 8 indicates complete independence in performing instrumental activities of daily living and a lower score suggests functional impairment, except when activities never performed by the older adult are omitted [21]. The Montreal Cognitive Assessment (MoCA), a brief screening tool, covers six cognitive domains with a score range of 0-30. A cut-off point of 24 on the MoCA is identified as an indicator of possible dementia in Mexican validation [22]. The Epworth Sleepiness Scale (ESS) assesses sleep propensity in eight different situations, with scores ranging from 0 to 24. A total score less than 10 is considered normal, 10-12 indicates marginal sleepiness, and above 12 suggests excessive sleepiness [23]. The Benzodiazepine Dependence Questionnaire (BDEPQ-MX) is a 30-item self-report tool assessing benzodiazepine experience over the past month on a Likert scale. A cut-off point of 23 points is identified for dependence [24].

The research, approved by the thesis and ethics committees of INPRFM, followed a comparative, observational, cross-sectional, retrolective, and homodemic study design. Recruitment involved advertising and inviting eligible patients over 60 from the outpatient clinic. Informed consent was obtained, and assessments were conducted using the mentioned tools for benzodiazepine consumers. If additional assistance was required to enhance the participant's comprehension, two witnesses were utilized to ensure understanding. Ethical considerations were adhered to minimize risk, following health research regulations and the Declaration of Helsinki. Approved by the INPRFM Ethics Committee, the study prioritized informed consent, participant awareness, confidentiality, and anonymity.

Data were recorded in Microsoft Excel (Microsoft Corp., Redmond, WA)and analyzed using SPSS software (IBM Corp., Armonk, NY). Statistical analysis involved a descriptive examination of clinical and sociodemographic variables to define participants' consumption profiles. Group comparisons utilized t-tests or chi-square tests, depending on the nature of the data.

## Results

In this study, we included a total of 162 participants who visited the outpatient clinic from June 2022 to March 2023. The demographic breakdown showed a predominance of female participants at 72.2% (n=117), with males comprising 27.8% (n=45). The average age was 66.88 ± 6.79 years (range 60-90 years). Regarding marital status, a little over half (53.1%, n=86) were married, 40.1% (n=65) were single, and a small fraction were either in a free union (6.2%, n=10) or divorced (0.6%, n=1). The average education level spanned 9.20 ± 4.22 years, and most participants were either engaged in household activities (52.5%, n=85) or employed (25.9%, n=42), with a smaller percentage being retired (18.5%, n=30) or unemployed (3.1%, n=5).

In our descriptive analyses comparing benzodiazepine (BZD) users and non-users, various differences were observed. Within the BZD group, there were fewer men (23.5%) compared to the non-BZD group (32.1%), and the average ages were closely matched, with the BZD group averaging 66.75 years and the non-BZD group at 67.01 years. The average educational level was slightly lower in the BZD group (8.86 years) compared to the non-BZD group (9.54 years), and marital status varied, with a higher percentage of married individuals in the non-BZD group (61.7% vs. 44.4% in the BZD group). It is crucial to note that these observed differences in sociodemographic characteristics were not statistically significant.

Clinically, depressive disorders were more prevalent in the BZD group (79%) compared to the non-BZD group (61.7%). It is noteworthy that this difference in prevalence did not achieve statistical significance. In terms of medical diagnoses, conditions such as diabetes and hypertension showed a similar prevalence across both groups. The BZD group exhibited a significantly higher incidence of multiple psychiatric comorbidities, with 64.2% having two comorbidities and 4.9% having three, in contrast to 46.9% and 6% in the non-BZD group, respectively. This disparity was found to be statistically significant, evidenced by a Pearson chi-square value (x^2^) of 24.102 and a p-value of <0.001. Additionally, tobacco use was observed to be more prevalent in the BZD group (21%) compared to the non-BZD group (4.9%), yet, similarly, this difference in prevalence was not statistically significant.

Clinimetric outcomes indicated significant differences between the groups in cognitive function, sleepiness, and daily living activities (Table 1). The MoCA total scores were lower in the BZD group (21.07) compared to the non-BZD group (23.31). The Epworth Sleepiness Scale scores were higher in the BZD group (6.21), indicating more sleepiness, as opposed to the non-BZD group (4.17). Similarly, Lawton's Index scores, which measure daily living activities, were lower in the BZD group (6.56) compared to the non-BZD group (7.52).

**Table 1:** Comparison Between Groups on Categorical Variables The statistical analysis conducted utilized the chi-squared test. A p-value of 0.005 or lower was deemed significant, with an alpha level set at 0.05. Abbreviations: BZD = Benzodiazepine; IADL = Instrumental Activities of Daily Living; ADL = Activities of Daily Living

Variables	BZD Use Group (n = 81)	Non-BZD Use Group (n = 81)	χ²	p
Possible dementia	60 (74.1%)	43 (53.1%)	7.704	0.006
Excessive daytime sleepiness	14 (17.3%)	4 (4.9%)	6.250	0.120
Reduced functionality (IADL/ADL)	39 (48.1%)	18 (22.2%)	11.937	<0.001

The analysis of benzodiazepine usage characteristics revealed that the majority of BZD prescriptions were for their anxiolytic effects (80.2%) and hypnotic effects (19.8%). The average age at the onset of BZD use was around 55 years, with a considerable duration of use averaging over 4000 days. Psychiatrists were the most common prescribers of BZDs.

When analyzing the differences among consumers of BZD, it was found that those with possible dementia had a higher average daily dose of diazepam equivalents (23.09 mg) compared to those without possible dementia (15.46 mg) (Table 2). A significant proportion of BZD users (66.66%) showed dependence, with this number rising to 90% among those with possible dementia. Additionally, a lower level of education was observed in participants with possible dementia.

**Table 2:** Differences in Participants in the BZD Consumption Group "Mean" refers to the average scores obtained in the MoCA assessment, with their standard deviations provided in parentheses. Similar to the first table, this analysis employed the Student's t-test for statistical evaluation. A p-value of 0.005 or lower was considered significant, with an alpha level established at 0.05. Abbreviations: BZD = Benzodiazepine

Variable	Possible dementia (n=60; mean/SD)	No possible dementia (n=21; mean/SD)	t	p
Diazepam equivalent dose (mg)	23.0900 (19.4830)	15.4600 (11.2490)	-2.170	0.017
Days of vonsumption	4320.7000 (3517.9140)	3779.5200 (3987.5560)	-0.586	0.280
BZD dependence	0.7167 (0.45442)	0.5238 (0.51177)	-1.620	0.068
Years of education	7.9300 (3.9990)	11.5200 (4.1060)	3.517	<0.001

Overall, the study's findings highlight significant differences in demographics, clinical characteristics, and clinimetric outcomes between older adults who are BZD consumers and those who are not, underscoring the impact of benzodiazepine use on various aspects of health and daily functioning.

## Discussion

Prescription of benzodiazepines in older adults

The prescription of benzodiazepines (BZDs) in older adults is a critical issue, as their use can be “potentially inappropriate” and requires rational administration. There is limited information in our population regarding the effects of BZDs on functionality, cognition, and sleep. This study contributes valuable insights into the cognitive, daytime somnolence, and functional impacts of BZD use in older adults.

Sociodemographic data

In our sample, a higher percentage of BZD users were women (76.5%), aligning with international studies indicating a range between 67.4% and 74% [25]. The non-BZD group demonstrated a slightly higher level of education (average 9.54 years) compared to the BZD group (average 8.86 years), consistent with findings that lower education levels are associated with higher BZD consumption rates, especially in Latin America [19].

Clinical data

Depression was the most prevalent psychiatric disorder in both groups, paralleling previous findings [17]. Similarly, systemic arterial hypertension was the most common medical condition, mirroring recent international studies [21, 17].

Comparative instrument analysis

The MoCA screening tool, used to assess cognitive function, revealed a significant difference in scores between the groups (p<0.001), with the non-BZD group scoring higher (23.31±4.008) than the BZD group (21.07±4.367). This result is consistent with a study from China [26]. Furthermore, using a cut-off point of 24 points on MoCA, identified as an indicator of possible dementia [22], 74.1% of the BZD group scored below this threshold, compared to 53.1% in the non-BZD group.

The Epworth Sleepiness Scale scores were higher in the BZD group, indicating increased daytime somnolence, a condition characterized by the inability to maintain wakefulness and alertness during the day [27]. This finding aligns with research by Jaussent et al., which reported higher somnolence in older adults using hypnotics [28].

Regarding the Lawton Index, assessing daily living activities like phone use or financial management, 48.1% of the BZD group showed reduced functionality compared to 22.2% in the non-BZD group, mirroring findings by Carrière et al. [13].

BZD users comparison

Around 74.1% of the BZD-consuming older adults scored below or equal 24 on MoCA, a threshold for positive possible dementia screening. The average diazepam-equivalent dose in this group was 23.09 mg, compared to 15.46 mg in the non-possible dementia group, similar to findings by Chen et al. [29]. However, the duration of BZD use did not show a significant impact.

Higher education levels were observed in BZD users who scored above 24 on MoCA, comparable to the results reported by Guo et al. [26]. An inverse correlation was found between diazepam equivalent dose and performance on the naming subtest of MoCA, suggesting that higher doses may impair linguistic and perceptual-visual skills, an early symptom in Alzheimer’s disease [29, 30].

BZD dependence

The study found that 66.6% of BZD users showed dependence according to the BDEPQ-MX, with 90% of these having a positive screening for possible dementia. This is concerning, considering the risks associated with BZD use in older adults, including cognitive decline, delirium, falls, fractures, and car accidents, regardless of the type of BZD prescribed [28]. Nonetheless, for patients with severe generalized anxiety disorder, discontinuation might lead to further functional deterioration, emphasizing the clinician’s responsibility in prescribing and potentially tapering BZDs based on evidence [31, 32].

Strengths and limitations

The study's strengths include an adequate sample size, the use of standardized instruments validated in our country, and the selection of participants from outpatient services, providing broader representation. It's the largest study conducted at INPRFM assessing cognition, daytime somnolence, functionality, and BZD dependence in older adults.

However, limitations include the absence of tools to assess acute affective symptoms or anxiety exacerbation, possibly skewing results. The single-time measurement also limits the depth of the findings, suggesting the need for repeated measurements for a more robust generalization. Furthermore, it is important to consider that the chronicity of benzodiazepine use was not precisely measured or compared across groups. Additionally, the evaluation was not conducted for age groups divided by years of education, which could be a potential confounding factor.

## Conclusions

In conclusion, the findings of this study suggest that the consumption of benzodiazepines may be associated with possible dementia, as well as increased drowsiness and functional dependence compared to non-users. Additionally, a direct relationship was found between the equivalent dose of benzodiazepines and the potential onset of dementia. These results highlight the importance of considering the potential adverse effects of these substances on individuals' health and the need for adequate surveillance and regulation in their prescription and use.
